# THE ROLE OF FISTULOCLYSIS IN THE TREATMENT OF PATIENTS WITH ENTEROATMOSPHERIC FISTULAS

**DOI:** 10.1590/0102-672020210002e1605

**Published:** 2021-10-18

**Authors:** Marcelo Augusto Fontenelle RIBEIRO-JUNIOR, Daniel Dante YEH, Samara de Souza AUGUSTO, Yasmin Garcia Batista ELIAS, Paola Rezende NÉDER, Cássia Tiemi Kawase COSTA, Andressa Daniel MAURÍCIO, Salomone Di SAVERIO

**Affiliations:** 1 Discipline of Trauma and General Surgery, Pontifical Catholic University of São Paulo, Sorocaba, SP, Brazil; 2 Division of Trauma and Surgical Critical Care, Miller School of Medicine, University of Miami, Miami, FL, USA; 3 Medical College, University Santo Amaro Medical School, São Paulo, SP, Brazil; 4 Department of General Surgery, University of Insubria, Regione Lombardia, Varese, Italy

**Keywords:** Intestinal fistula, Malnutrition, Nutritional support, Enteral nutrition, Parenteral nutrition, Fístula intestinal, Desnutrição, Suporte nutricional, Nutrição enteral, Nutrição parenteral

## Abstract

**Background:**

Enterocutaneous fistulas represent a connection between the gastrointestinal tract and adjacent tissues. Among them, there is a subdivision - the enteroatmospheric fistulas, in which the origin is the gastrointestinal tract in connection with the external environment through an open wound in the abdomen. Due to the high output in enterocutaneous fistulas, the loss of fluids, electrolytes, minerals and proteins leads to complications such as sepsis, malnutrition and electrolyte derangements. The parenteral nutrition has its secondary risks, and the fistuloclysis, that consist in the infusion of enteral feeding and also the chyme through the distal fistula, represents an alternative to the management of these patients until the definitive surgical approach.

**Aim:**

To evaluate the current evidence on the fistuloclysis technique, its applicability, advantages and disadvantages for patients with high output fistulas.

**Method:**

A systematic literature search was conducted in May 2020 with the headings “fistuloclysis”, “chyme reinfusion” and “succus entericus reinfusion”, in the PubMed, Medline and SciELO databases. **Results:** There were 29 articles selected for the development of this narrative synthesis, from 2003 to 2020, including reviews and case reports.

**Conclusion:**

Fistuloclysis is a safe method which optimizes the clinical, nutritional, and immunological conditions of patients with enteroatmospheric fistulas, increasing the chances of success of the reconstructive procedure. In cases where the definitive repair is not possible, chances of reducing or even stopping the use of nutrition through the parental route are increased, thus representing a promising modality for the management of most challenging cases.

## RESUMO

**Racional:**

As fístulas enterocutâneas representam uma conexão entre o trato gastrointestinal e os tecidos adjacentes. Dentre elas, há uma subdivisão - as fístulas enteroatmosféricas, em que a origem é o trato gastrointestinal em conexão com o meio externo por meio de uma ferida aberta no abdômen. Devido ao alto débito nas fístulas enterocutâneas, a perda de fluidos, eletrólitos, minerais e proteínas levam a complicações como sepse, desnutrição e desequilíbrios eletrolíticos. A nutrição parenteral tem seus riscos secundários, e a fistuloclise, que consiste na infusão de nutrição enteral e também do quimo pela fístula distal, representa uma alternativa no manejo desses pacientes até a abordagem cirúrgica definitiva.

**Objetivo:**

Avaliar as evidências atuais sobre a técnica de fistuloclise, sua aplicabilidade, vantagens e desvantagens para pacientes com fístulas de alto débito.

**Método:**

Foi realizada uma busca sistemática da literatura em maio de 2020 com os títulos “fistuloclysis”, “chyme reinfusion” e “succus entericus reinfusion”, nas bases de dados PubMed, Medline e SciELO.

**Resultados:**

Foram selecionados 29 artigos para o desenvolvimento desta síntese narrativa, no período de 2003 a 2020, incluindo revisões e relatos de caso.

**Conclusão:**

A fistuloclise é um método seguro que otimiza as condições clínicas, nutricionais e imunológicas dos pacientes com fístulas enteroatmosféricas, aumentando as chances de sucesso do procedimento de reconstrução. Nos casos em que o reparo definitivo não é possível, aumentam as chances de reduzir ou mesmo interromper o uso da nutrição pela via parental, representando uma modalidade promissora para o manejo dos casos mais desafiadores.

## INTRODUCTION

Fistulas are abnormal communications between two organs, an organ and skin, or an organ and a wound. When enteric, enterocutaneous fistulas represent a connection between the gastrointestinal tract and the skin. Another complex kind of fistula is represented by the enteroatmospheric fistulas (EAF), in which the origin is the gastrointestinal (GI) tract in connection with the external environment through an open abdomen^2^ ^,^ ^3^ ^,^ ^7^ ^,^ ^9^ ^,^ ^11^ ^,^ ^12^ ^,^ ^14^ ^,^ ^18^ ^,^ ^20^ ^,^ ^21^ ^,^ ^23^ ^,^ ^26^ ^,^ ^28^. In the case of EAF, most of them are complications of intestinal perforations, abdominal operations such as resection of the colon and/or rectum, surgical re-explorations, anastomotic fistulas and complex abdominal traumas. However, between 15-25% of enterocutaneous fistulas arise spontaneously in patients with inflammatory bowel disease (most commonly Crohn’s disease); exposure to radiation; oncological diseases; distal intestinal obstruction; and intestinal infections such as tuberculosis, amoebiasis, and typhoid fever^2^ ^,^ ^9^ ^,^ ^11^ ^,^ ^12^ ^,^ ^14^ ^,^ ^16^ ^,^ ^18^ ^,^ ^21^ ^,^ ^26^.

Intestinal failure can occur on patients with fistulas and it was defined by the European Society for Clinical Nutrition and Metabolism as “the reduction of gut function below the minimum necessary for the absorption of macronutrients and/or water and electrolytes, such that intravenous supplementation is required to maintain health and/or growth”. In case of enterocutaneous fistulas, the intestinal failure is classified as type 2, and defines as a prolonged acute condition, often in metabolically unstable patients, requiring complex multidisciplinary care, and intravenous supplementation over periods of weeks or months. The current gold standard therapy indicated until the surgical reestablishment of intestinal continuity is home parenteral nutrition^21^.

The mortality rate of these patients is variable, from 6-30%, depending of output, demonstrating the complexity of such complication^3^. The most lethal impacting factor is represented by the high output EAF, since the loss of fluids, electrolytes, minerals and proteins leads to complications such as sepsis, malnutrition and electrolyte derangements^2^ ^,^ ^3^ ^,^ ^7^ ^,^ ^9^ ^,^ ^11^ ^,^ ^12^ ^,^ ^14^ ^,^ ^16^ ^,^ ^17^ ^,^ ^23^ ^,^ ^25^ ^,^ ^27^ ^,^ ^28^. Fistulas can be classified by different criteria, as shown in Table 1 ^6^.

**TABLE 1:** Classification of enteroatmospheric fistulas (EAFs)^6^

Localization	Proximal	Stomach, duodenum, jejunum or proximal ileum
	Distal	Distal ileum or colon
Daily output	Low	<200 ml/24 h
	Moderate	200 - 500 ml/24 h
	High	>500 ml/24 h
Location at the open abdomen	Superficial	Drains through the wound of the abdominal cavity
	Deep	Drains intestinal contents into the abdominal cavity
Number of fistulas	Single	One single fistula
	Multiple nearby fistulas	Two or more fistulas close together
	Multiple distant fistulas	Two or more distant fistulas

The management of high-output (>500 ml/24 h) fistulas still represents a challenge for the multidisciplinary team due to metabolic impairment. The initial approach aims to provide systemic support for the proper functioning of vital organs, infection source control, management of the surgical wound, electrolyte balance maintenance, and the maintenance of the gastrointestinal tract function. Initially, the treatment aims to reverse the inflammatory pattern and encourage spontaneous closure of the lesion^2^ ^,^ ^7^ ^,^ ^10^ ^,^ ^11^ ^,^ ^14^ ^,^ ^15^ ^,^ ^17^ ^-^ ^19^ ^,^ ^22^ ^,^ ^23^ ^,^ ^25^ ^-^ ^28^ ^,^ ^30^.

In the case of EAFs, it is unlikely that spontaneous resolution of moderate and high output fistulas will occur^5^. The treatment becomes long-term to maintain the patient’s clinical conditions and later definitive correction with reestablishment of GI continuity^4^ ^,^ ^7^ ^,^ ^9^ ^,^ ^11^ ^,^ ^12^ ^,^ ^15^ ^,^ ^18^ ^,^ ^22^ ^-^ ^24^ ^,^ ^27^. Although many studies recommend a surgical approach after three months, currently it is recommended to wait around 12 months for the best results to allow for adequate nutritional stabilization, optimal infectious control and a better intra-abdominal condition with fewer and softer adhesions during the surgical re-approach^14^ ^,^ ^23^.

Since definitive treatment is not feasible in the short term, it is absolutely necessary to improve patient’s nutritional status in order to achieve clinical stability. With persistent enteric flow through EAF, there is a significant loss of fluids associated with loss of nutrients; low daily caloric intake, and high energy demand resulting from the inflammatory process, will result in an intense catabolism and subsequent malnutrition^9^ ^-^ ^12^ ^,^ ^24^ ^,^ ^28^ ^,^ ^30^.

In the face of one of the greatest complications of these patients, the treatment of choice for many years was the use of total parenteral nutrition (TPN), associated avoiding oral or enteral nutrition and administration of drugs such as octreotide to reduce intrinsic intestinal secretion and decrease motility. The aim at this stage is to reverse catabolism, establish the rest of the gastrointestinal tract and reduce the output of the EAF. Even though it has been the main nutritional approach adopted, TPN has negative repercussions on treatment and requires proper care. Among the complications are liver dysfunction, catheter infections, and hyperglycemia^2^ ^-^ ^4^ ^,^ ^8^ ^-^ ^11^ ^,^ ^14^ ^,^ ^16^ ^-^ ^18^ ^,^ ^22^ ^,^ ^24^ ^,^ ^26^ ^-^ ^28^.

As the nutrition of the patient with an EAF has an extremely important role in the management of the case, for the reduction of morbidity and mortality, care must be taken in defining the treatment. The exclusive parenteral route, even though it is the most widely used option, carries relevant secondary risks and high costs to the patient^3^ ^,^ ^7^ ^,^ ^8^ ^,^ ^10^ ^,^ ^11^ ^,^ ^14^ ^,^ ^15^ ^,^ ^17^ ^,^ ^18^ ^,^ ^20^ ^,^ ^24^ ^-^ ^28^.

The alternative nutritional management to TPN is the use of enteral nutrition (EN). Except in cases contraindicated due to intestinal discontinuity, difficulty to insert the catheter to provide enteral access, or intolerance, this route should be considered whenever possible to replace TPN^2^ ^,^ ^18^. According to the study published by Ortiz et al^14^, there is consensus among the studies published in the preference of EN over TPN when both are available.

By using the enteral route, which is more physiological in principle, the mesenteric perfusion is stimulated by mesenteric postprandial blood flow, the structural and functional integrity of the GI tract mucosa is increased, bacterial adhesion to the intestinal epithelial cells is prevented, with greater stimulation of IgA secretion and less local inflammatory response. These factors enhance the trophism of the local mucosa, in the long-term will provide better results in the definitive surgery for the reconstruction of intestinal transit^11^ ^-^ ^14^ ^,^ ^16^ ^,^ ^26^ ^,^ ^28^ ^,^ ^30^.

EN provides greater benefits to the patient’s clinical condition, also reducing the secondary risks of TPN in its replacement. However, according to Bradasawi et al^2^, substitution for enteral nutrition in patients with high-output fistula is of little use in maintaining the metabolic state and in managing the patient’s complications. EN could potentially exacerbate malnutrition and delay the definitive correction due to an augmentation of the fistula output^9^ ^,^ ^12^ ^,^ ^22^ ^,^ ^28^.

A technique described by Teubner et al^24^, termed fistuloclysis, uses the distal limb of the fistula itself as a route for nutritional administration, that can include EN and the proximal fistula output. This approach is an alternative to the management of these patients who receive exclusive nutritional support via parenteral route, thereby avoiding the secondary complications previously exposed.

Some studies showed that the administration of the proximal fistula output, containing the succus entericus or chyme, can be beneficial to these patients. The advantages of chyme reinfusion depend mainly on the level of the fistula, as the effluent can vary in its content. It can be rich in salivary amylase, gastric pepsin, pancreatic enzymes and bile. In this way, meet the needs of macronutrients, micronutrients, mineral salts, water, electrolytes and bile salts^14^ ^,^ ^17^. Acordding to Picot et al^17^ chyme reinfusion corrected the intestinal failure by restoring intestinal absorption, allowing parenteral nutrition weaning in 91% of patients. The intestinal losses were reduced by 85% (p<0.001) and the number of patients with output higher than 1200 ml/24 h decreased from 155 to 9 (p<0.0001). With this procedure, the number of patients who had liver tests abnormalities decreased from 87 to 51% (p<0.001). It was also associated with the improvement of nutritional status and Nutritional Risk Index that means, mean weight gain, body mass index and plasma albumin. Thereby chyme reinfusion contributes to improve nutritional status and to reduce plasma liver tests abnormalities. As well, two studies reported another advantage of it favoring economic outcome, demonstrating savings in total healthcare cost per patient^20^ ^,^ ^25^.

The objective of this review was to evaluate the current evidence on the fistuloclysis technique, its applicability, advantages and disadvantages for patients with high output fistulas.

## METHOD

### Search strategy

A systematic literature search was conducted in May 2020 with the headings “fistuloclysis”, “chyme reinfusion” and “succus entericus reinfusion”, in the PubMed, Medline and SciELO databases, all papers between 2003 and 2020 in English and Spanish related to the topic were reviewed. No study type filters were used.

### Screening and synthesis of evidence

**FIGURE 1:**
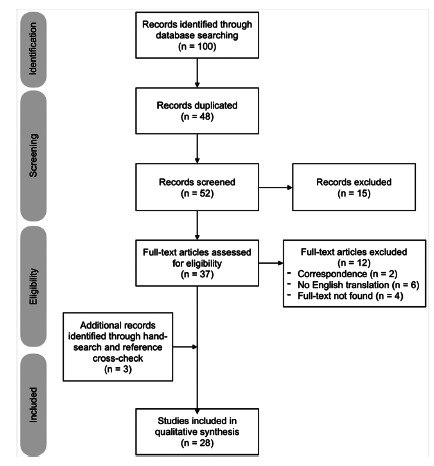
PRISMA flow diagram for the identification and screening of articles

The database search resulted in 100 records. After the exclusion of 48 duplicate articles, 52 articles were analyzed by title and summary, excluding those 15 dealing with other subjects. From 37 articles read in full, 12 were excluded for different reasons and three others were included by hand-search and reference cross-check. Finally, 28 articles were selected for this review (Figure 1).

### Data analysis

Given the heterogeneous nature of the included texts, a quantitative meta-analysis was not possible, and the focus of this review was a narrative qualitative synthesis of available data. In view of the types of retrieved literature (detailed below), a formal appraisal of study quality was not conducted.

## RESULTS

Twenty-nine articles were selected from 2003 to 2020, including review papers, case reports, case series, cohort studies and clinical trials. There were only two prospective studies and all other ones were retrospectives^1^ ^-^ ^4^ ^,^ ^7^ ^-^ ^30^ (Table 2).

**TABLE 2:** Characteristics of the included studies

Author	Year	Study	Study design
Appleton et al^1^	2019	Case Report	Retrospective
Bradasawi et al^2^	2015	Review	Retrospective
Bhat et al^3^	2019	Systematic review	Retrospective
Carlson et al^4^	2003	Review	Retrospective
Du Toit A et al^7^	2014	Review and case report	Retrospective
Farrer et al^8^	2015	Cohort	Retrospective
Ham et al^9^	2007	Review and case report	Retrospective
Kaushal et al^10^	2004	Review	Retrospective
Kumpf et al^11^	2017	Review	Retrospective
Lloyd et al^12^	2006	Review	Retrospective
Niu et al^13^	2019	Case Report	Retrospective
Ortiz et al^14^	2017	Review	Retrospective
Peer et al^15^	2008	Case Report	Retrospective
Pflug et al^16^	2013	Case Report	Retrospective
Picot et al^17^	2017	Cohort	Prospective
Polk et al^18^	2012	Review	Retrospective
Sathyanarayana et al^19^	2005	Case Report	Retrospective
Sharma et al^20^	2020	Clinical trial	Prospective
Sica et al^21^	2007	Review and case report	Retrospective
Slater et al^22^	2009	Review	Retrospective
Stein SL^23^	2019	Review	Retrospective
Teubner et al^24^	2004	Serie of cases	Retrospective
Thibault et al^25^	2016	Review	Retrospective
Willcutts et al^26^	2015	Case Report	Retrospective
Wright et al^27^	2013	Case Report	Retrospective
Wu et al^28^	2014	Cohort case series	Retrospective
Ye et al^29^	2013	Case Report	Retrospective
Yuan et al^30^	2011	Cohort	Retrospective

## DISCUSSION

The management of the fistula effluent of high output fistulas is essential for obtaining the benefits of enteral nutrition. To maintain this nutritional route, the technique used must collect the content drained by the fistula, ensuring the protection of the adjacent tissue so that there is no further damage to the skin and avoiding chemical dermatitis. The technique, defined as fistuloclysis, uses the distal fistula as an enteral entry port for possible administration of enteral formulas and gastrointestinal secretions^2^ ^,^ ^5^ ^,^ ^7^ ^,^ ^8^ ^,^ ^10^ ^,^ ^21^ ^,^ ^23^ ^,^ ^25^ ^,^ ^26^. Wu et al^4^ and Farrer et al^28^ described the fistuloclysis technique as a safe procedure in the challenging management of high-output fistulas.

To determine the feasibility of fistuloclysis, it is necessary to perform a procedure known as a fistulogram. Contrast is injected into the distal fistula through a catheter and then a radiological study is performed to monitor the flow of the contrast. Thus, it is possible to identify the length of the distal GI tract, the presence or absence of obstructions, and to confirm the exact location of the fistula. For the patient to be classified as a candidate for the use of the technique, it is necessary that the fistula orifice be permeable to the entry of a catheter and no distal obstruction is present. Associated with the conditions for the procedure, the patient must be hemodynamically stable, with no active infections, and with no possibility of spontaneous resolution of the fistula in the near future^2^ ^,^ ^10^ ^,^ ^15^ ^,^ ^16^ ^,^ ^18^ ^,^ ^21^ ^,^ ^26^ ^,^ ^27^. According to Teubner et al^5^, when the intestinal mucosa is in contact with the skin or interspersed with granulation tissue, spontaneous resolution of the fistula is unlikely.

**FIGURE 2:**
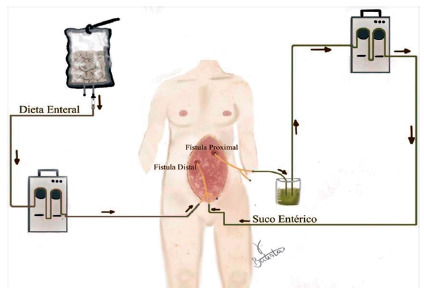
Fistuloclysis technique: collection of enteric fluid in a non-sterile reservoir, use of an infusion pump (or use of a 60ml syringe for aspiration and reinfusion, as shown in figure 2) and infusion of the chyme using a three-way Foley catheter

Although very promising, the technique is not widely practiced. For this reason, there is no standardization of the materials used to perform fistuloclysis. Even if there is variation, the mechanism must be performed in the same way to establish an extracorporeal continuity of the circulation of the gastrointestinal content. There should always be a means of collecting the enteric content of the afferent proximal fistula, and creating a continuity, promoting reinfusion in the distal fistula through a catheter^5^ ^,^ ^9^ ^,^ ^11^ ^,^ ^16^ ^-^ ^18^ ^,^ ^20^ ^,^ ^22^ (Figures 2, 3 and 4 and video https://youtu.be/WLLOVe_yDLU). 

**FIGURE 3:**
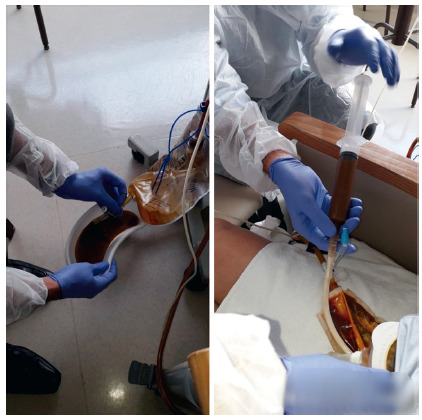
Bedside collection of chime for reinfusion into the distal fistula

Picot et al^17^ and Thibault et al^25^ report the need to feed on pasty textures to avoid catheter obstruction as inconveniences inherent to the technique in regard to quality of life. However, in our practice we recommend the infusion of liquids (EN and chime) through the distal feeding catheter and the patients can eat by mouth (liquids and pasty textures food) as they wish. Regarding possible complications of fistuloclysis, there is escape of the collected effluent content and corrosion of the mucosa and skin that need to be rehabilitated, as well as displacement of the tube by peristalsis.

**FIGURE 3:**
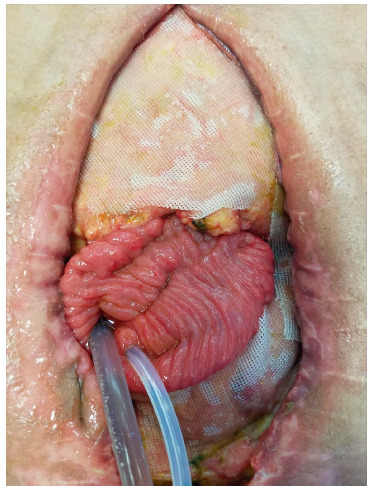
Open abdomen with proximal silicone catheter for collection of the chyme and the distal one for subsequent reinfusion and also infusion of the enteral nutrition diet

With the implementation of this technique in cases of high output fistulas, the collected effluent content (composed of salivary amylase, gastric pepsin, pancreatic enzymes and bile) promotes the restoration of intestinal absorption and these enzymes act directly in the recovery malnourished patient, as well as normalizing the enterohepatic circulation. The treatment, in turn, responds with important nutritional improvement, being able to completely replace TPN by enteral nutrition. Thus, the clinical repercussions secondary to it are corrected and avoided^3^ ^,^ ^4^ ^,^ ^8^ ^,^ ^13^ ^-^ ^15^ ^,^ ^17^ ^,^ ^19^ ^-^ ^22^ ^,^ ^28^ ^,^ ^29^. Other advantages are the reduction of fistula effluent, improvements in electrolyte balance, and the lower cost of treatment compared to the use of TPN^2^ ^,^ ^3^ ^,^ ^14^ ^,^ ^20^ ^,^ ^28^ ^,^ ^29^. When establishing the enteral nutrition route using the fistuloclysis technique, another benefit includes positive impact on liver function, reduction in the risk of bacterial translocation, and consequently prevention of sepsis^12^ ^,^ ^17^ ^,^ ^20^ ^,^ ^27^ ^,^ ^28^ ^,^ ^30^. According to Picot et al^17^, some of the forms of liver damage in the patients treated conventionally with TPN are the use of GI tract-inhibiting drugs, and the excessive growth of bacteria in the small intestine (bacterial overgrowth). As the distal loops remain at rest due to the absence of intraluminal stimulation of nutrients and motility, intestinal permeability increases, bacteria adhere to the intestinal epithelium and release their toxins, promoting translocation to the liver. In the proximal loops, the process is even easier because there is no absorption of nutrients and by the use of proton pump inhibiting and anti-motility drugs.

According to Ortiz et al^14^, the operational success rate of fistuloclysis when the team is specialized is around 70-92%. According to Picot et al^17^ and Thibault et al^25^ in 59 (28%) patients on their case series, chyme reinfusion was feasible at home in selected patients after training and education, where they must have acquired total autonomy for chyme reinfusion and basic stoma care. The median duration of home chyme reinfusion was 36-40 days. As a result, only a few were readmitted for minor problems, and none had to stop chyme reinfusion or go back to parenteral nutrition. As a conclusion they understand more studies are needed including a greater number of patients and centers to demonstrate safety and clinical benefits of home chyme reinfusion.

After the recommended minimum period of 12 months, surgery for definitive repair can be scheduled. According to Teubner et al^5^, when the maintenance of the patient’s clinical conditions is due to EN, performed in association with the fistuloclysis, there is an important improvement in the function of the intestinal mucosa barrier. In this way, the atrophy of the intestinal mucosa is prevented or limited after exclusive treatment for TPN and rest of the GI tract, and in reconstructive surgery, the caliber and thickness of the tissue to be sutured is much firmer. Fistuloclysis, although offering significant advantages for the patient, is rarely applied in medical practice. Much is due to limitations of the patient himself, who often refuses the idea of ​​reinfusion of enteric content after its excretion and the fact that the mechanism used to make gastrointestinal extracorporeal circulation feasible is not very pleasant. For the multidisciplinary team, the low adherence is due to the high demand required for the application of the technique as well as lack of familiarity^2^ ^,^ ^3^ ^,^ ^14^ ^,^ ^16^ ^,^ ^20^ ^,^ ^27^. One side effect was reported for the first time, by Appleton N et al^1^, was the pneumatosis intestinalis secondary to fistuloclysis. Some studies have reported another side effects as disadvantages of the technique, such as diarrhea, vomiting, nausea, pain and abdominal distention^5^ ^,^ ^7^ ^-^ ^10^ ^,^ ^14^ ^,^ ^20^ ^,^ ^28^. According to Picot et al^17^ and Thibault et al^25^, these described side effects would be present anyway after definitive reconstruction of intestinal transit if there was no enteral nutrition by fistuloclysis.

## CONCLUSION

Fistuloclysis is a safe method which optimizes the clinical, nutritional, and immunological conditions of patients with EAF, increasing the chances of success of the reconstructive procedure. In cases where the definitive repair is not possible, chances of reducing or even stopping the use of nutrition through the parental route are increased, thus representing a promising modality for the management of most challenging cases.
